# Enhancing *Pisum sativum* growth and symbiosis under heat stress: the synergistic impact of co-inoculated bacterial consortia and ACC deaminase-lacking *Rhizobium*

**DOI:** 10.1007/s00203-024-03943-3

**Published:** 2024-04-04

**Authors:** Roukaya Ben Gaied, Imed Sbissi, Mohamed Tarhouni, Clarisse Brígido

**Affiliations:** 1 Laboratory of Pastoral Ecosystems and Promotion of Spontaneous Plants and Associated Micro-Organisms, Institute of Arid Lands, University of Gabes, Medenine 4119, Tunisia; 2 MED – Mediterranean Institute for Agriculture, Environment and Development, Universidade de Évora, Pólo da Mitra, Ap. 94, Évora, 7006-554 Portugal; 3 MED-Mediterranean Institute for Agriculture, Environment and Development & CHANGE-Global Change and Sustainability Institute, Institute for Advanced Studies and Research, Universidade de Évora, Pólo da Mitra, Ap. 94, Évora, 7006-554 Portugal

**Keywords:** Nodule-associated endophytes, Root exudation, Bacterial consortia, Abiotic stress, Nitrogen fixation, Nodulation

## Abstract

The 1-aminocyclopropane-1-carboxylate (ACC) deaminase is a crucial bacterial trait, yet it is not widely distributed among rhizobia. Hence, employing a co-inoculation approach that combines selected plant growth-promoting bacteria with compatible rhizobial strains, especially those lacking ACC deaminase, presents a practical solution to alleviate the negative effects of diverse abiotic stresses on legume nodulation. Our objective was to explore the efficacy of three non-rhizobial endophytes, *Phyllobacterium salinisoli* (PH), *Starkeya* sp. (ST) and *Pseudomonas turukhanskensis* (PS), isolated from native legumes grown in Tunisian arid regions, in improving the growth of cool-season legume and fostering symbiosis with an ACC deaminase-lacking rhizobial strain under heat stress. Various combinations of these endophytes (ST + PS, ST + PH, PS + PH, and ST + PS + PH) were co-inoculated with *Rhizobium leguminosarum* 128C53 or its Δ*acdS* mutant derivative on *Pisum sativum* plants exposed to a two-week heat stress period.

Our findings revealed that the absence of ACC deaminase activity negatively impacted both pea growth and symbiosis under heat stress. Nevertheless, these detrimental effects were successfully mitigated in plants co-inoculated with Δ*acdS* mutant strain and specific non-rhizobial endophytes consortia. Our results indicated that heat stress significantly altered the phenolic content of pea root exudates. Despite this, there was no impact on IAA production. Interestingly, these changes positively influenced biofilm formation in consortia containing the mutant strain, indicating synergistic bacteria-bacteria interactions. Additionally, no positive effects were observed when these endophytic consortia were combined with the wild-type strain. This study highlights the potential of non-rhizobial endophytes to improve symbiotic performance of rhizobial strains lacking genetic mechanisms to mitigate stress effects on their legume host, holding promising potential to enhance the growth and yield of targeted legumes by boosting symbiosis.

**Supplementary Information:**

The online version contains supplementary material available at 10.1007/s00203-024-03943-3.

## Introduction

The importance of legume plants derives from their unique capacity to form a mutualistic relationship with rhizobia, a bacterial species that can fix atmospheric nitrogen (Oldroyd 2013). However, this symbiotic interaction is hardly maintained under abiotic stresses, such as heat stress (Zahran 1999). Heat stress affects the legume-*rhizobium* symbiosis, either directly by limiting the growth of the microsymbiont and/or indirectly by modulating the physiological and biochemical state of the host plant (Sita et al. 2017).

Some rhizobial strains exhibit a variety of plant growth-promoting characteristics (PGP) (Brígido et al. 2017), which can, directly or indirectly, improve crop adaptation to extreme environments. Among these abilities, particular attention was given to 1-aminocyclopropane-1-carboxylate (ACC) deaminase enzyme for its importance in the modulation of internal levels of ethylene in legume roots and in the nodulation process (Nascimento et al. 2016). Ethylene is a gaseous phytohormone involved in different steps of plant growth and development. While under optimal conditions, ethylene is produced in small quantities, under environmental stresses, its production increases significantly in plant organs (Abeles 1992), which can cause leaf senescence, reduction in chlorophyll content, inhibition of root elongation and nodulation, and sometimes plant death (Djanaguiraman and Prasad 2010). ACC deaminase-producing rhizobia can alleviate the detrimental effects of ethylene stress on legume tissues, by simply degrading ACC (ethylene precursor) into α-ketobutyrate and ammonia utilized by soil microbes as a source of carbon and nitrogen. Thus, promoting legume growth and symbiosis under stress conditions (Nascimento et al. 2012a, b; Brígido et al. 2013; Saghafi et al. 2019). However, only approximately 1–10% of rhizobial strains naturally possess ACC deaminase in the fields (Duan et al. 2009). Additionally, most rhizobia usually produce very low levels of ACC deaminase compared to other non-rhizobial PGP bacteria (Nascimento et al. 2016). These low levels of ACC deaminase in rhizobia are generally not sufficient to effectively decrease the high levels of ethylene accumulated in plants due to abiotic stress (Glick et al. 2007; Nascimento et al. 2016). Moreover, rhizobial strains lacking ACC deaminase activity showed not only low levels of competitiveness for nodule formation but also an accelerated nodule senescence (Alemneh et al. 2020). For instance, the inoculation of mung bean plants with the mutant strain *Sinorhizobium* sp. BL3 lacking ACC deaminase resulted in a decrease of strain competitiveness, the formation of smaller nodules, and high bacteroids mortality compared to nodules induced by the wild-type strain (Tittabutr et al. 2015). Hence, revealing the important role of ACC deaminase in both rhizobia competitiveness and nodule function.

In the light of these challenges, a lot of attention has been addressed to nodule-associated endophytes (non-rhizobial endophytes) and their potential role in legume-*rhizobium* symbiosis. This unique bacterial population represents a great resource of PGP bacteria (Martínez-Hidalgo and Hirsch 2017). In fact, many different genera among which *Pseudomonas*, *Phyllobacterium*, *Mycobacterium* and *Starkeya* (Zakhia et al. 2006; De Meyer et al. 2015) showed various PGP traits in vitro (Pandya et al. 2015), including those associated with legumes native to arid lands (Hnini et al. 2023). These isolates showed a better resilience to abiotic stress, which has been referred to their natural adaptability to harsh environments (Soussi et al. 2016; Alsharif et al. 2020). Furthermore, the use of non-rhizobial endophytes well adapted to harsh environments along with compatible rhizobia revealed positive effects on the growth and nodulation of legumes, especially those exposed to stress conditions (Brígido et al. 2019a; Bessadok et al. 2020; Paço et al. 2020; Knežević et al. 2021; Flores-Duarte et al. 2022).

Considering the increasing frequency and intensity of extreme weather events, this study aims to investigate the potential of three non-rhizobial endophytes, isolated from root nodules of wild legumes grown in arid regions, to improve the symbiotic performance of rhizobia lacking ACC deaminase activity under heat stress. We tested the ability of these strains to overcome the loss of ACC deaminase functionality in *Rhizobium leguminosarum* Δ*acdS* mutant derivative, in comparison with its wild-type strain *R. leguminosarum* 128C53, a microsymbiont of *Pisum sativum* L. Overall, our study aims to enrich current knowledge, providing valuable information about the negative impacts of the lack of ACC deaminase activity on *rhizobium*-pea symbiosis under high temperature events, introducing non-rhizobial endophytes isolated from legumes native to arid regions as a potential tool to mitigate the adverse effects on both legume growth and symbiosis under heat stress.

## Materials and methods

### Bacterial strains, growth conditions and compatibility test

Three non-rhizobial endophytes (IRAMC:0104, IRAMC:0020, and IRAMC:0040) from our bacterial collection, which was obtained from root nodules of wild legumes growing in various bioclimates in Tunisia (Sbissi et al., *Unpublished data*), were chosen for this study. These strains were isolated from root nodules of three legumes, *Calobota saharae*, *Calicotome villosa*, and *Coronilla scorpioides*, which were growing in three different Saharan and arid regions of Tunisia, as previously described by (Zakhia et al. 2006). Based on the 16S rRNA gene analysis (*Sbissi et al. Unpublished data*), IRAMC:0104, IRAMC:0020 and IRAMC:0040 isolates were assigned to *Phyllobacterium salinisoli* (referred as PH, NCBI accession number OR179644), *Pseudomonas turukhanskensis* (referred as PS, NCBI accession number OR179645), and *Starkeya* sp. (referred as ST, NCBI accession number OR179646), respectively.

The rhizobial strains *Rhizobium leguminosarum* 128C53 (WT) and its *ΔacdS* mutant derivative (MT) (Ma et al. 2003) were used as pea (*Pisum sativum* L.) microsymbionts. All bacterial strains were routinely grown at 28 °C on yeast extract mannitol (YEM) medium (Vincent 1970), unless expressed elsewhere. Kanamycin was added to the YEM medium for the *ΔacdS* knockout mutant at a concentration of 50 µg·mL^− 1^.

The compatibility between our non-rhizobial endophytic strains with rhizobium WT and MT strains was assessed following the protocol outlined by (Paço et al. 2020). Briefly, all strains (both rhizobia and non-rhizobia) were individually cultured in YEM liquid medium at 28 °C with agitation for 48 h. Following incubation, bacterial suspensions were standardized to an optical density (OD_600nm_) of 1. Subsequently, 100 µl of each microsymbiont suspension (WT or MT) were spread onto YEM agar plates and incubated at 28 °C for 1 to 2 h. Afterward, 10 µl of each non-rhizobial bacterial culture were spotted in triplicate onto the agar plates previously inoculated with the rhizobial strain. The plates were then incubated at 28 °C and monitored for 48 to 96 h. The absence of an inhibition zone indicates compatibility between the microsymbiont and the non-rhizobial endophyte.

### Heat tolerance assay

Heat tolerance of both rhizobial and non-rhizobial endophytes strains was evaluated by measuring the optical density at 540 nm (OD_540_) of the bacterial growth in liquid medium under different heat conditions. Bacterial cells were standardized to an initial OD_540_ of 0.3 for rhizobia and 0.1 for non-rhizobial endophytes prior to heat stress or shock treatment. Bacterial suspensions were exposed to continuous heat stress at 40 °C, while for heat shock, the cells were incubated at 45 °C for 15 min, followed by continuous growth at 28 °C. Continuous bacterial growth at 28 °C was considered as control condition for each bacterial isolate. Three replications of each treatment were performed.

Rhizobial bacterial growth was evaluated in YEM liquid medium at 6 h intervals during 52 h, whereas non-rhizobial endophytes were grown in tryptic soy broth (TSB, Liofilchem) and OD_540_ measurements were taken at intervals of < 10 h for 28 to 48 h.

### In vitro screening of plant growth promoting (PGP) activities

The ability of non-rhizobial endophytes to solubilize phosphate and synthesize siderophores was assessed in Pikovskaya and Chrome Azurol S plates, respectively, as previously described (Brígido et al. 2019b). A clearance zone observed around the colonies, characterized by a transparent zone for phosphate solubilization and an orange zone for siderophore production, indicated a positive result for both plant growth-promoting (PGP) traits. The phosphate solubilization index (PSI) and siderophore production index (SPI) were determined using the formula (halo diameter + colony diameter)/colony diameter).

The evaluation of the 1-aminocyclopropane-1-carboxylate (ACC) deaminase activity of the bacterial endophytes was performed under free-living conditions according to the method described by (Brígido et al. 2015). The strains *Pseudomonas putida* UW4 (Glick 1995) and *Mesorhizobium ciceri* LMS1 (Brígido et al. 2012) were used as a positive and negative controls, respectively, in the ACC deaminase assay. The ability of bacterial endophytes to produce IAA was estimated as previously described (Ben Gaied et al. 2024).

### Plant growth assay under heat stress

To assess the influence of non-rhizobial bacterial endophytes on the symbiotic performance of rhizobial WT and MT strains with their host plant under heat stress, two separate plant growth assays were conducted in a growth chamber. These assays utilized gnotobiotic systems with pea plants (*Pisum sativum* L.) subjected to heat stress conditions. We chose rhizobial WT and its MT strain to examine the effects of ACC deaminase activity absence on the interaction between rhizobia and their host plants under heat stress. Additionally, we investigated whether co-inoculation with non-rhizobial endophytic bacteria could mitigate the impacts of ACC deaminase activity absence in MT-pea symbiosis.

The inoculum of rhizobial and endophytic strains was prepared as described previously (Brígido et al. 2019a), by growing separately in YEM medium at 28 °C with agitation for 48 h. Bacterial cultures were washed twice with sterile saline solution, and then resuspended in the same solution adjusted to an optical density of 0.9 for rhizobial strains and of 0.5 for non-rhizobial endophytes at 600 nm.

Pea seeds were surface sterilized with ethanol (96–100%) for 5 min, followed by an immersion in 0.2% of HgCl_2_ prepared in sterile distilled water for 5 min followed by ten times rinses with sterile distilled water. To induce pre-germination, the seeds were placed in 0.75% agar plates and incubated in the dark at 28 °C for 4–5 days. After seed germination, the seedlings were transferred to sterilized plastic pots previously filled with a sterile sand:vermiculite 1:2 (v/v) mixture. Pea seedlings (one per pot) were immediately inoculated with 2 mL of bacterial suspension (containing single or bacterial consortia). The treatments were prepared as follows: (i) plants inoculated only with WT or MT microsymbiont, (ii) plants inoculated with one of the rhizobial WT or MT strain and a consortium containing endophytic bacterial isolates. Four non-rhizobial endophytes consortia were used along with rhizobial WT or MT: (i) *Starkeya* sp. and *Phyllobacterium salinisoli* (ST + PH), (ii) *Pseudomonas turukhanskensis* and *P. salinisoli* (PS + PH), (iii) *Starkeya* sp. and *P. turukhanskensis* (ST + PS), and (iv) *Starkeya* sp., *P. turukhanskensis* and *P. salinisoli* (ST + PS + PH). Five pot replicates were used for each treatment.

All pots were placed in a growth chamber with a photoperiod of 16 h/24°C for the day cycle and 8 h/18°C for the night cycle and 65% humidity as standard conditions (Paço et al. 2020). Following two weeks of growth, a heat stress event was initiated, consisting of consecutive cycles of temperatures ranging from 30 to 35 °C for 16 h during the day cycle, with intervals of 30 °C for 6 h, 32 °C for 6 h, and 35 °C for 4 h, while maintaining a temperature of 20 °C for 8 h during the night cycle, all under 60% humidity. This heat stress regimen was repeated daily for two consecutive weeks. Plants were irrigated with a nitrogen-free nutritive solution (Broughton and Dilworth 1971) alternatively with distilled sterilized water, whenever necessary. Uninoculated plants watered with a nitrogen-free nutrient solution (Negative control) were used to control cross contamination in both plant trials. The pea plants were harvested after seven weeks of growth.

At the end of the experiment, the shoots were separated from the roots and the number of nodules was recorded. The shoot (SDW), root (RDW) and nodule (NDW) dry weights were measured. To determine the chlorophyll content of plant shoots, a 0.1 g portion of fresh leaves was macerated in 90% acetone, centrifuged at 8000 g for 5 min and the absorbance was measured using a UV-spectrophotometer at 645 and 663 nm. Chlorophyll a, chlorophyll b and total chlorophyll contents were determined according to the procedure of Arnon (1949).

### Extraction and analysis of root exudates composition

Given the common effects of heat stress on plant metabolism and, consequently, root exudates composition, pea root exudates (RE) were collected from pea plants grown under control (NRE) or heat stress conditions (HRE) for further analysis. Root exudates were extracted under control conditions, as described by (Srivastava et al. 1999). Under heat stress, seedlings were grown for 5 days at 28 °C followed by 5 days at 30 °C. Under both conditions, seedlings were individually placed in 50 ml conical tubes containing 10 ml of distilled sterilized water, with 10 replicates per condition. After 7 days of cultivation, root exudates were collected, and filter-sterilized using a 0.22 μm pore filter membrane. Aliquots of 100 µl were plated on Luria Bertani medium and incubated for 48 h at 28 °C to check for eventual contamination. After filter-sterilization, part of the root exudates was lyophilized for analysis of the composition of phenolic compounds (phenolic acids and flavonoids) and the other part was stored at -80 °C until further use in bioassays.

Phenolic compounds analysis was performed as previously described by (Ben Gaied et al. 2024) using a quadrupole mass spectrometer: LC-MS-2020 (Shimadzu, Kyoto, Japan) equipped with an electro-nebulization ionization source (ESI) in negative mode. Briefly, a volume of 20 µl of exudates per sample was injected in an Inert sustain C18 column (GL Sciences Japan) (150 mm x 3 mm, 3 μm). The spectral data were monitored in SIM (Selected Ion Monitoring) mode and were analysed using the Shimadzu Lab solutions LC-MS software. High-purity nitrogen served as both nebulizer and auxiliary gas. The identity of phenolic compounds was determined by matching the obtained retention times and mass spectra to chemical standards of > 98% purity obtained from Sigma Chemical Co. (St Louis, MO, USA). Analysis involved three independent replicates per treatment.

### Effect of pea root exudates on Biofilm and IAA production

To evaluate the potential impact of *P. sativum* root exudates on plant-bacteria interaction and bacteria-bacteria interactions, in vitro quantification of biofilm formation and IAA production by individual strains or in consortia was performed on free-living cell cultures with and without exposure to pea root exudates collected under normal (NRE) or heat stress (HRE) conditions.

IAA production was performed according to (Brígido et al. 2019b), both with and without pea root exudates under heat stress (HRE). Overnight cultures of rhizobial (WT and MT) and non-rhizobial (ST, PS, and PH) strains were adjusted to an OD_600_ nm of 0.05 in YEM medium supplemented with tryptophan (Trp) at a final concentration of 250 µg.ml^− 1^ or with Trp 250 µg.ml^− 1^ + 100 µl of HRE (OD_280_ nm = 0.08). Bacterial cultures were then incubated at 28 °C for 48 h, with three replicates for each sample. IAA production was estimated using an IAA standard curve as described earlier (Brígido et al. 2019b).

The quantification of biofilm formation was performed in microtiter PVC plates as previously described in (Torres et al. 2021), with slight modifications. In brief, bacterial cultures of individual and combined strains were standardized to an initial OD_600_ nm of 0.5 in YEM. To evaluate the effect of root exudates composition on biofilm formation, 30 µl of root exudates from control (NRE) or from heat stress (HRE) conditions were mixed with 150 µl of bacterial cell suspensions. Plates were covered, sealed with parafilm, and incubated at 28 °C for 72 h without agitation. Three replicates were used per treatment under each condition. Biofilm formation was quantified at 565 nm and normalized by cell growth (OD_600_ nm).

### Statistical analysis

Statistical analyses were performed using IBM SPSS statistics version 21 (IBM Corp., Armonk, N.Y., USA). Data from the plant trial and root exudates composition were analysed using an independent samples t-test (*p* < 0.05 and *p* < 0.01). One-way analysis of variance (ANOVA) and the post-hoc test Tukey were used to evaluate the significant differences among treatments and bacterial species in terms of plant growth promoting features and biofilm production (*p* < 0.05). Effects of root exudates and bacterial species on biofilm formation and IAA production was studied using two-way ANOVA. A heatmap displaying the identified phenolic compounds in pea root exudates collected under each condition was created using MetaboAnalyst 6.0 (Xia et al. 2009), following square root normalization of the data.

## Results

### Bacterial tolerance to heat stress

**Fig. 1:**
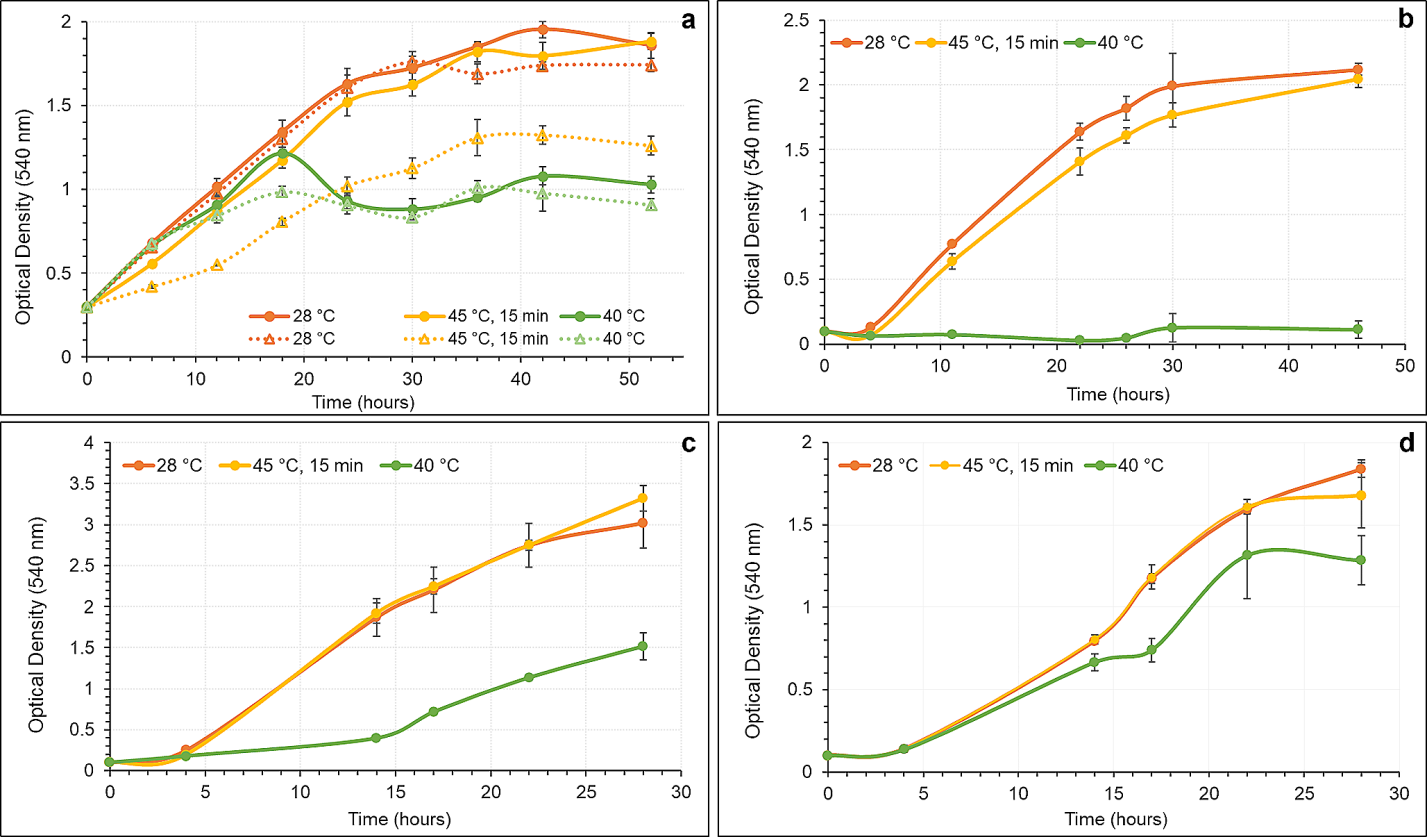
Growth curves of rhizobial and non-rhizobial endophytes under different heat conditions (continuous growth at 28 °C (control) and 40 °C (heat stress); and at 45 °C for 15 min followed by 28 °C (heat shock)) during 28–52 h. (**a**): Growth curves of rhizobial strains (continuous lines correspond to the growth of *R. leguminosarum* strain 128C53 (WT), dotted lines correspond to the growth of *ΔacdS* mutant derivative strain (MT); (**b**): Growth curves of *P. turukhanskensis* (PS); (**c**): Growth curves of *Starkeya* sp. (ST); (**d**): Growth curves of *Ph. salinisoli* (PH); error bars correspond to the standard deviation

To investigate the heat tolerance of the non-rhizobial and rhizobial strains, their growth in liquid medium was evaluated at both control (28 °C) and high temperatures (40 °C and heat shock at 45 °C for 15 min). The growth rates varied substantially among the bacterial strains (Fig. 1). Under control conditions (28 °C) and heat stress (40 °C), no significant variations in the growth rates of the rhizobial wild-type (WT) and the *ΔacdS* mutant (MT) strains were found (Fig. 1a). In contrast, when exposed to heat shock (45 °C for 15 min), the MT strain showed a lower growth rate than the WT, suggesting that the ACC deaminase is involved in the heat stress adaptation. In fact, the WT strain was able to recover from heat shock while the MT strain did not, resulting in a substantial decrease in its growth rate.

*P. turukhanskensis* (PS) was the most vulnerable strain among non-rhizobial endophytes, particularly to continuous heat stress (Fig. 1b) as no growth was observed for 48 h. Nevertheless, its growth rate after heat shock was only slightly lower compared to control conditions. In contrast, both *Starkeya* sp. (ST) and *Ph. salinisoli* (PH) isolates exhibited similar growth rates under control and heat shock conditions (Fig. 1c, d), suggesting that these strains are genetically well-suited to withstand heat shocks. Under continuous heat stress, both strains presented lower growth rates compared to control conditions, with ST growth being more affected than PH.

### PGP activities of endophytic strains

The non-rhizobial bacterial endophytes were evaluated for their plant growth promotion (PGP) potential, namely the synthesis of indoleacetic acid (IAA), siderophores production, phosphate solubilization and ACC deaminase activity.

Different PGP traits have been associated to the endophytic strains (Table 1). The PH strain exhibited the highest levels of phosphate solubilisation and IAA production. Both PS and ST were unable to solubilize phosphate, but both produced more siderophores than PH strain. None of the endophytes exhibited ACC deaminase activity under free-living conditions (Table 1).

**Table 1:** In vitro plant growth-promoting features of non-rhizobial endophytes

Bacterial isolates	PGP features			
	ACC deaminase activity	IAA production (µg/ml)	Siderophores production index (SPI)	Phosphate solubilization index (PSI)
*Ph. salinisoli* (PH)	-	42.5 ± 2.76 ^a^	1.44 ± 0.05^b^	2.65 ± 0.15^a^
*P. turukhanskensis* (PS)	-	34.48 ± 6.34^ab^	2.53 ± 0.11^a^	-
*Starkeya* sp. (ST)	-	24.04 ± 4.37^b^	2.47 ± 0.13^a^	-

(-) Negative or no activity, the SPI and PSI were calculated as (halo diameter + colony diameter)/ colony diameter); Data represents the mean and standard deviation of three independent biological replicates. Significant differences are indicated with different letters in the same column by Tukey test (*p<0.05*)

### Effect of non-rhizobial endophytes on pea growth, symbiotic rhizobium-pea relationship and photosynthesis in plants submitted to a heat stress event

Two plant trials were conducted in growth chambers to study the impact of non-rhizobial bacterial endophyte co-inoculation on rhizobium-pea symbiotic interaction in the absence of ACC deaminase activity. Four consortia, each containing different combinations of non-rhizobial bacterial endophytes (ST + PH, PS + PH, ST + PS, and ST + PS + PH), were tested on pea plants exposed to heat stress, alongside the compatible pea microsymbiont *R. leguminosarum* 128C53 (WT) or its Δ*acdS* mutant derivative (MT) strain.

**Fig. 2:**
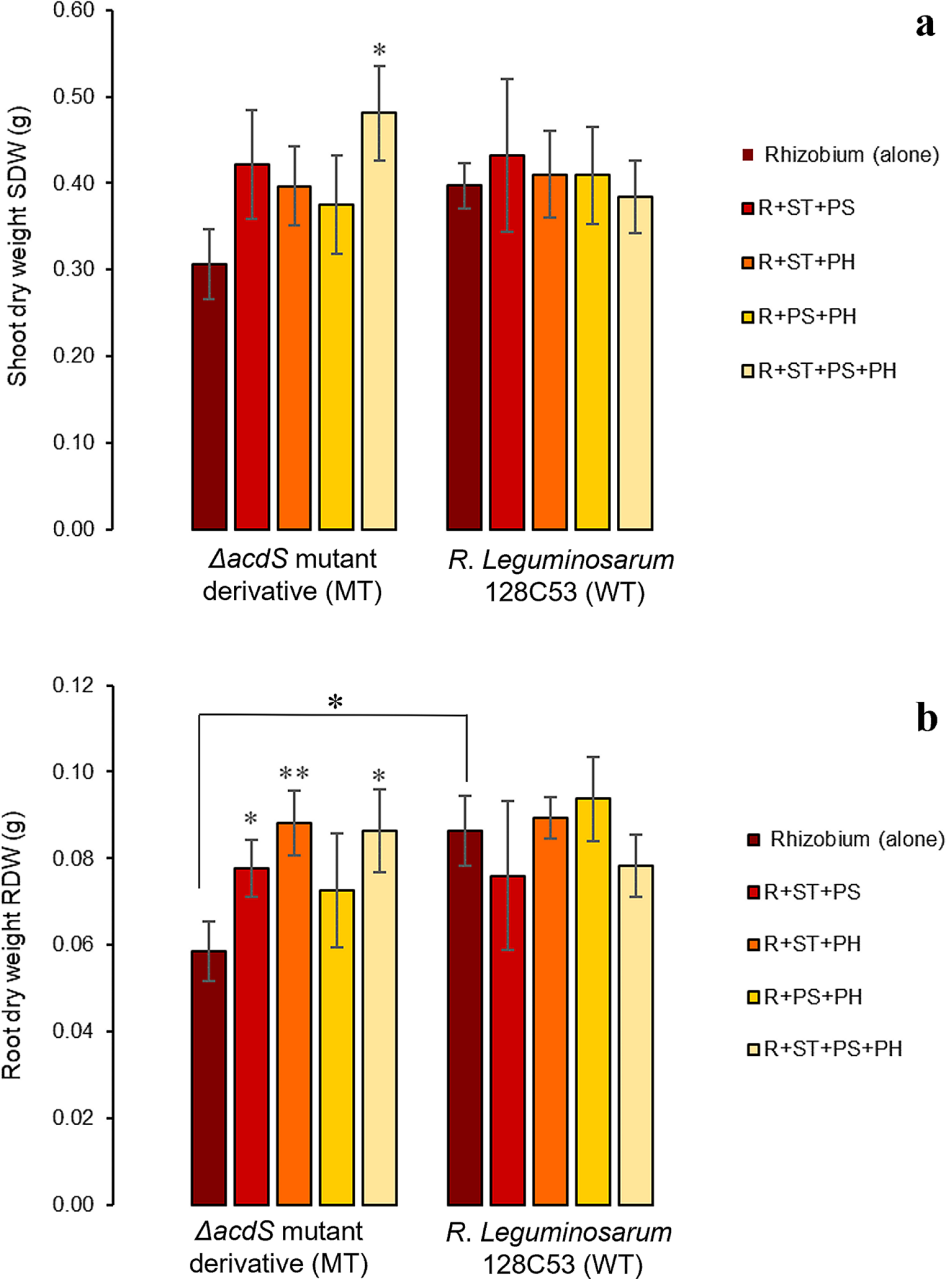
Effect of co-inoculating non-rhizobial endophytes on various plant parameters of pea grown under heat stress event. Data are means of two independent trials (*n = 10*); (**a**): shoot dry weight SDW; (**b**): root dry weight RDW; *Rhizobium* (alone) refers to *R. legumniosarum* 128C53 (WT) or Δ*acdS* mutant derivative (MT) inoculated alone; R denotes *R. legumniosarum* 128C53 (WT) or Δ*acdS* mutant derivative (MT) inoculated with non-rhizobial endophytes; ST represents *Starkeya* sp.; PS represents *P. turukhanskensis;* PH represents *Ph. salinisoli.* Error bars correspond to the standard error; * indicates statistical significance (*p* < 0.05); ** indicates significant differences (*p* < 0.01)

**Fig. 3:**
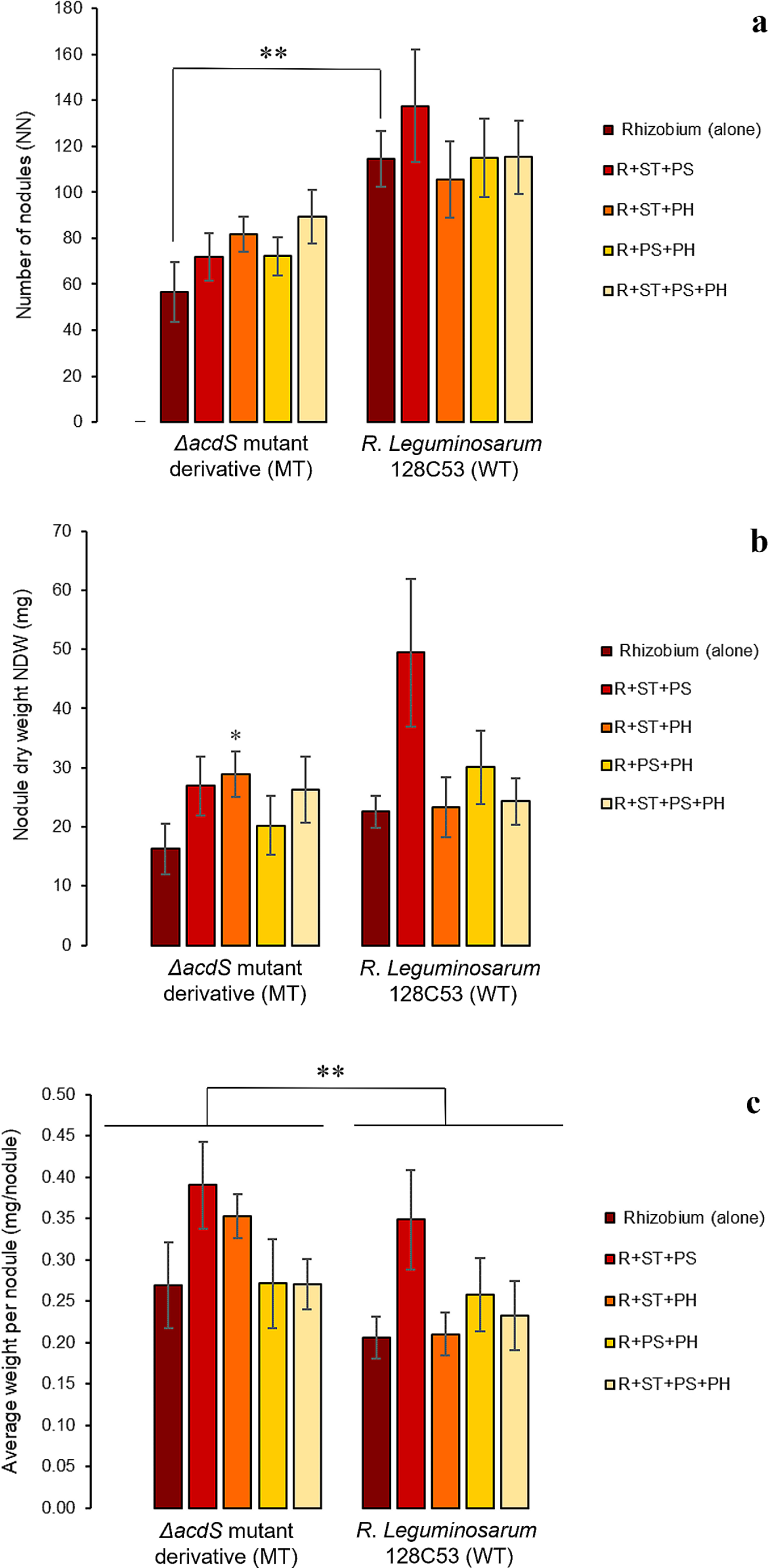
Effect of co-inoculation with non-rhizobial endophytes on symbiotic *Rhizobium*-pea interaction under a heat stress event. Data are means of two independent trials (*n = 10*); (**a**): number of nodules (NN), (**b**): nodule dry weight (NDW), and (**c**) average weight per nodule; *Rhizobium* (alone) refers to *R. legumniosarum* 128C53 (WT) or Δ*acdS* mutant derivative (MT) inoculated alone; R denotes *R. legumniosarum* 128C53 (WT) or Δ*acdS* mutant derivative (MT) inoculated with non-rhizobial endophytes; ST represents *Starkeya* sp.; PS represents *P. turukhanskensis;* PH represents *Ph. salinisoli.* Error bars correspond to the standard error; * indicates statistical significance (*p* < 0.05); ** indicates significant differences (*p* < 0.01)

As expected, plants inoculated solely with the MT strain exhibited a notable reduction in nodule formation and root dry weight (RDW), consequently affecting aboveground biomass to a lesser extent than plants inoculated solely with the WT strain. These findings underscore the adverse effects of ACC deaminase absence in rhizobium on both pea symbiosis and growth under heat stress (Figs. 2 and 3).

Interestingly, none of the tested consortia had significant beneficial effects on pea plants when co-inoculated with WT. Conversely, plants co-inoculated with both MT and non-rhizobial consortia displayed interesting synergistic interactions. Notably, there was a trend towards increased shoot dry weights (SDW) in plants co-inoculated with any of the consortia containing WT or MT, with the SDW of plants co-inoculated with MT + ST + PS + PH being statistically higher than those inoculated solely with MT (Fig. 2a). Moreover, the SDW of these plants surpassed that of plants inoculated or co-inoculated with WT (e.g. an increase of 20% compared to WT alone). This significant enhancement in aboveground biomass suggests that the synergistic interaction between MT and the endophytic strains within this consortium (ST + PS + PH) not only compensates for the absence of ACC deaminase but also enhances pea tolerance to heat stress.

The impact of co-inoculating plants with MT and any consortium containing non-rhizobial bacterial endophytes was more pronounced on RDW than on SDW when compared to plants solely inoculated with MT. All consortia containing non-rhizobial bacterial endophytes significantly enhanced RDW, except for the consortium containing PS + PH, when co-inoculated with MT. Notably, all consortia containing the ST strain exhibited a substantial promotion in RDW when co-inoculated with MT, indicating that the combination of strains within the consortium is necessary to observe significant synergistic effects in plant growth. For example, plants co-inoculated with different consortia containing the ST strain showed a significant increase in RDW ranging from 32 to 49% compared to plants inoculated solely with MT (Fig. 2b), reaching RDW values comparable to those obtained from plants singly inoculated with the WT strain.

Regarding nodulation abilities, the absence of ACC deaminase activity in the MT symbiont contributed to a significant reduction in the number of formed nodules (NN) (Fig. 3a). This decrease in the number of nodules was also reflected in nodule dry weight (NDW), where a reduction of around 26% in NDW was observed in plants single inoculated with MT compared to plants inoculated with WT strain (Fig. 3b). Nevertheless, a trend towards an increase in the NN produced by MT strain was observed in plants co-inoculated with any of the non-rhizobial bacterial endophytes, being the highest NN recorded in plants co-inoculated with the consortium MT + ST + PS + PH. This consortium led to a 40.34% increase in NN compared to plants inoculated with MT alone. Despite that none of these treatments reached the average NN produced by the WT strain, the NDW of those nodules was similar or higher than that of plants inoculated only by WT. Co-inoculation of pea with the ST + PH consortium and the MT strain contributed to a significant increase of 77.55% in NDW compared to plants inoculated with MT alone (Fig. 3b) and of 32% in relation to plants single inoculated with the WT strain. These results indicate that any of the consortia together with the MT strain was able to promote the formation of larger nodules, contributing to an increase in the total nodule dry weight compared to MT strain (Fig. 3c). In fact, those nodules formed by the mutant strain in the presence of the non-rhizobial bacterial endophytes were heavier than the ones formed in inoculated plants by WT strain. These results suggest that co-inoculation of MT with the non-rhizobial endophytes somehow compensated the lack of ACC deaminase activity in MT-pea symbiosis, probably on the complementarily of plant growth-promoting mechanisms along with the N_2_-fixing symbionts, which resulted in the improvement of the nodule development under heat stress.

**Fig. 4:**
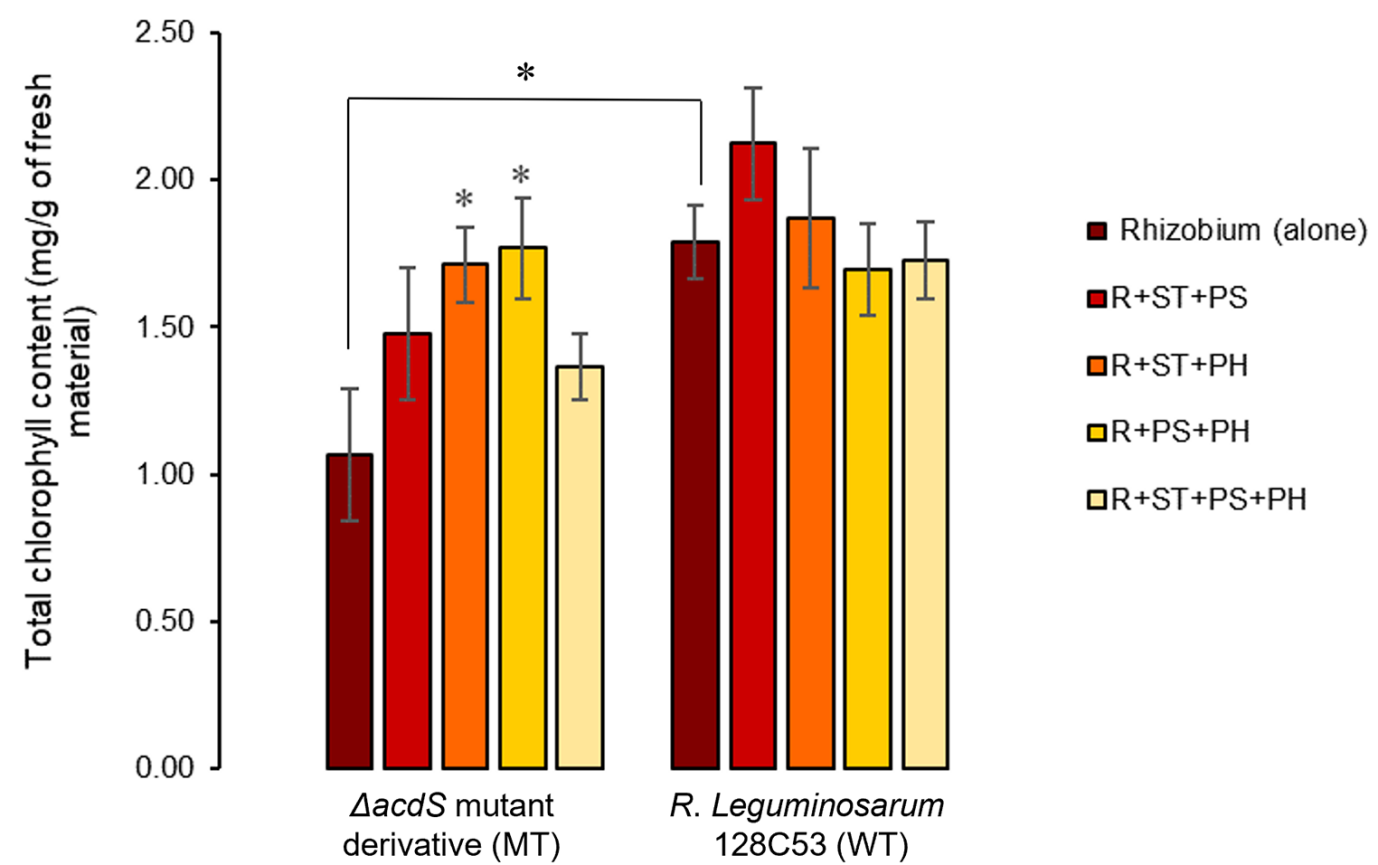
Effect of co-inoculation with non-rhizobial endophytes on pea photosynthesis submitted to heat stress (Total chlorophyll content (ChlTT)); (*n = 5* replicates per treatment); *Rhizobium* (alone) refers to *R. legumniosarum* 128C53 (WT) or Δ*acdS* mutant derivative (MT) inoculated alone; R denotes *R. legumniosarum* 128C53 (WT) or Δ*acdS* mutant derivative (MT) inoculated with non-rhizobial endophytes; ST represents *Starkeya* sp.; PS represents *P. turukhanskensis;* PH represents *Ph. salinisoli.* Error bars correspond to the standard error; * indicates statistical significance (*p* < 0.05)

Our results revealed that the absence of ACC deaminase activity in the MT strain negatively affected plant photosynthesis. Plants inoculated with the MT alone exhibited a 33% decrease in total chlorophyll content (ChlTT) compared to plants inoculated with the WT strain (Fig. 4). However, in the presence of endophytes, the photosynthetic capacity of pea plants inoculated with MT seemed to improve, resulting in a 40% increase in ChlTT content, regardless of the consortium employed. The two consortia, MT + ST + PH and MT + PS + PH, induced significant improvements of 60% and 65%, respectively, in ChlTT. In fact, the effect of inoculation with the bacterial consortium containing PS + PH on ChlTT was comparable to that of inoculation with the WT strain, suggesting that this combination of non-rhizobial endophytes had positive effects not only on MT-pea symbiosis but also on the plants’ ability to carry out photosynthesis under heat stress conditions. On the other hand, none of the consortia containing the WT strain showed a significant increase in ChlTT compared to the WT treatment.

### Composition of root exudates and effects on biofilm formation and IAA production

The phenolic composition of root exudates collected under both normal (NRE) and heat stress (HRE) conditions was analysed and compared to assess its potential impact on symbiosis and bacterial interactions. This comparison aimed to investigate whether changes in root exudate composition could explain the absence of positive effects observed in co-inoculated plants with WT and non-rhizobial endophytes, potentially influencing bacteria-bacteria communications and the symbiotic process.

**Fig. 5:**
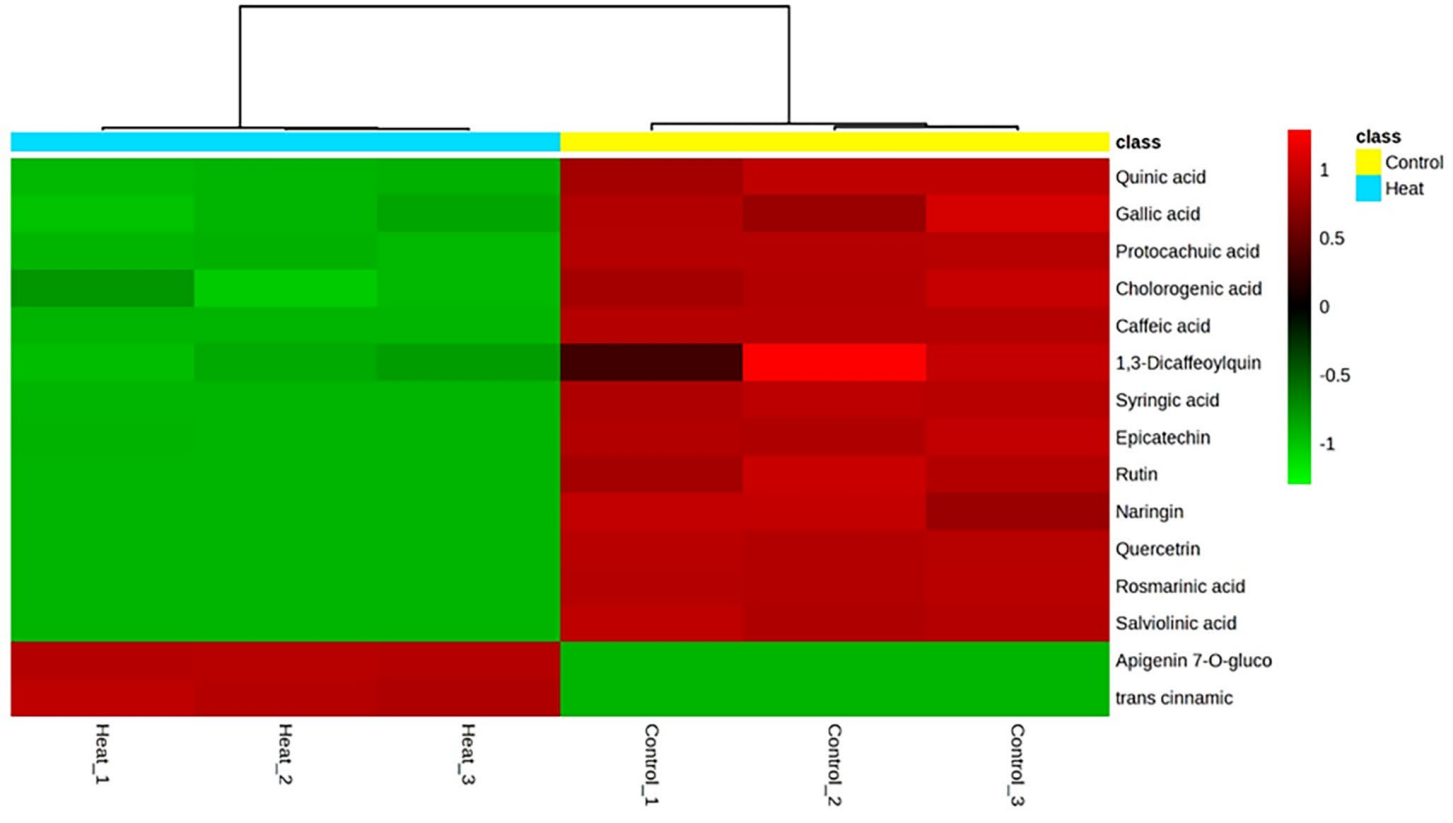
Heat map representing the abundance of phenolic compounds in pea root exudates exposed to normal and heat stress conditions. Results from three independent biological replicates for each treatment are displayed. “Control_” indicates root exudates collected under normal conditions, while “Heat_” indicates root exudates collected under heat stress. Numbers 1, 2, and 3 denote the biological replicate number within each treatment. The colour scale ranges (-1 to 1) from light green, indicating the absence or minimal abundance of phenolic compounds, to red, indicating the maximum abundance of phenolic compounds

Chemical analysis showed the presence of a variety of phenolic compounds (15 identified compounds, Table S1) with different concentrations in both samples. As expected, heat stress led to a significant alteration in composition and quantity of phenolic compounds compared to those collected under normal conditions (*p* < 0.01). For instance, rutin, naringin, quercetrin, rosmarinic acid and salviolinic acid compounds were only detected in root exudates collected under control conditions whereas Apigenin 7-O-glucoside and trans cinnamic were only identified in root exudates submitted to heat stress. Furthermore, all phenolic compounds commonly found in the two types of root exudates (control and heat stress) were found in significantly lower quantities in the HRE, proving that heat stress has a considerable impact on pea metabolism and exudation (Fig. 5).

To assess whether altered root exudate composition affects plant-bacteria or bacteria-bacteria interactions, IAA production and biofilm formation were measured in individual and/or in consortium with and without root exudates exposure.

**Fig. 6:**
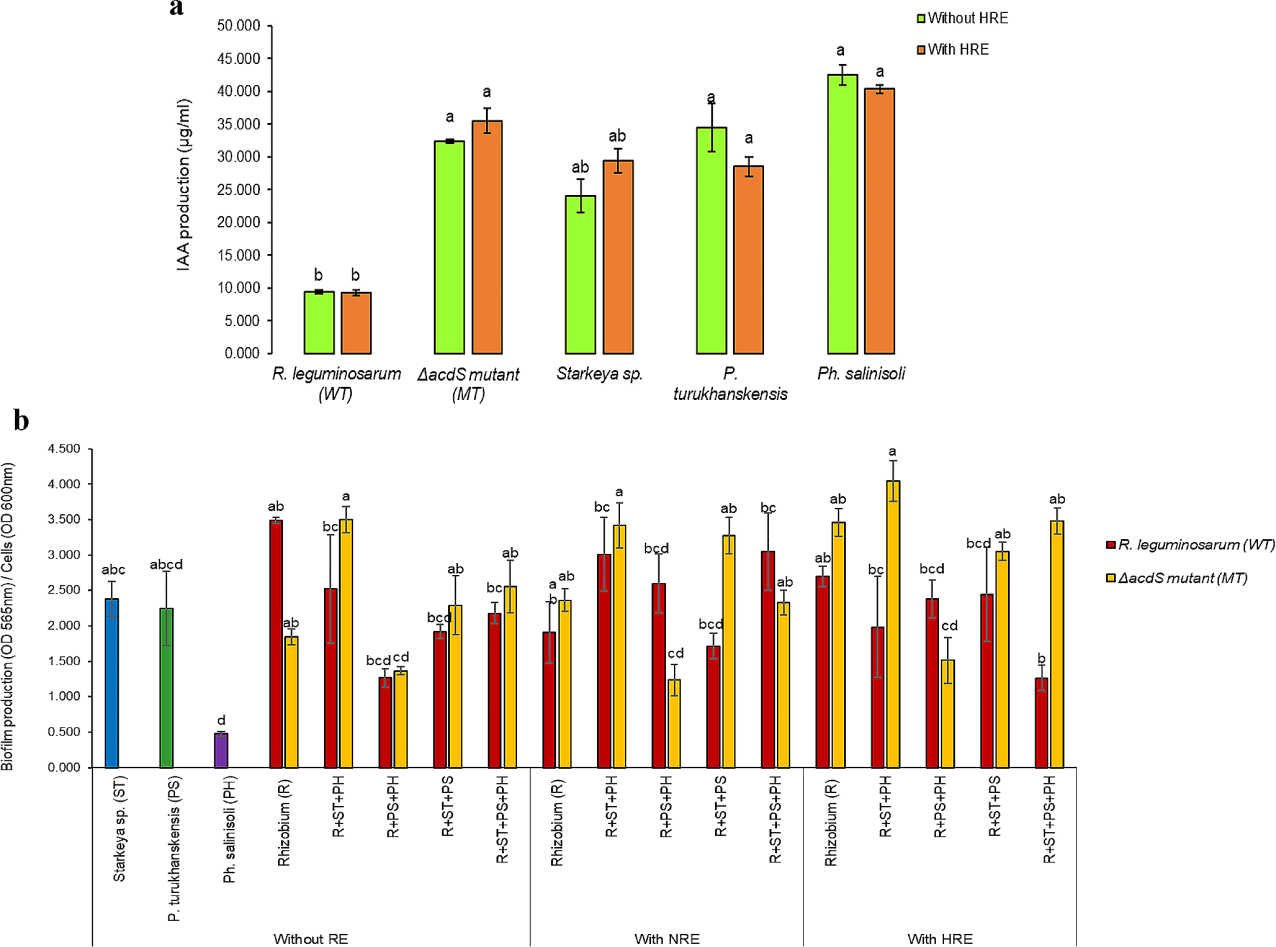
Effect of pea root exudates on IAA production and biofilm formation for both rhizobial and non-rhizobial endophytes. (**a**) IAA production with and without exposure to root exudates collected under heat (HRE) by each bacterial strain; (**b**) Biofilm production under the effect of root exudates (RE) collected under normal (NRE) or heat stress (HRE) conditions. The red bars represent data for *R. legumniosarum* 128C53 (WT) alone or in consortia with non-rhizobial endophytes while yellow bars correspond to data obtained for Δ*acdS* mutant derivative (MT) alone or in consortium. *Rhizobium* (R) refers to WT or MT inoculated alone; R denotes WT or MT inoculated with non-rhizobial endophytes; ST represents *Starkeya* sp.; PS represents *P. turukhanskensis;* PH represents *Ph. salinisoli.* Error bars correspond to the standard error. Different letters indicate statistical significance (*p* < 0.05)

Contrary to our expectations, the presence of root exudates collected under heat stress (HRE) had no effect on the production of IAA-like molecules by non-rhizobial endophytes (Fig. 6a). Nevertheless, bacterial species significantly influenced the levels of IAA production (F = 36.40, *p* < 0.001), with both PH and PS strains showing significantly higher IAA production than WT. More interestingly, MT strain exhibited significantly higher IAA levels than WT strain, suggesting a potential implication of the absence of ACC deaminase in the regulation of IAA biosynthesis pathways.

In the absence of root exudate stimuli, the rhizobial WT strain displayed the greatest amounts of biofilm formation, followed by *Starkeya* sp. (ST) and *P. turukhanskensis* (PS) (Fig. 6b). Notably, the MT strain, lacking ACC deaminase activity, showed a decrease in biofilm formation. When rhizobial strains were mixed with non-rhizobial endophytes, significant changes were observed in the production of biofilm. Consortia including the WT strain, for example, exhibited a tendency toward a reduction in biofilm production, while consortia including the MT strain had no effect. The MT + PS + PH consortium, however, increased biofilm development.

When rhizobial cells were exposed to root exudates, only the MT strain showed an increase in biofilm formation compared to control conditions (without root exudates). This increase was particularly pronounced in the presence of HRE. Similarly, when the MT strain was combined with any non-rhizobial endophytic consortia, there was an increase in biofilm formation, except for the PS + PH consortium. Interestingly, all consortia containing ST exhibited enhanced biofilm development, particularly in the presence of root exudates, either NRE or HRE. Indeed, the relationship between the ST + PH consortium and the MT strain significantly contributed to a substantial increase (*p* < 0.05) of biofilm production when exposed to root exudates. This finding suggests a positive interaction between the MT strain and the non-rhizobial endophyte ST. Conversely, the presence of root exudates did not affect the ability of the WT to form biofilm. Likewise, combining WT with various consortia did not lead to significant improvements in biofilm formation. These results indicate a lack of synergistic interaction between the rhizobial WT strain and non-rhizobial endophytes, consistent with findings from plant trials. Moreover, the statistical analysis revealed a significant impact of the interaction between bacterial species and root exudates on biofilm development, particularly evident in cells exposed to HRE, which contrasts with the findings from the IAA results (F = 3.541, *p* < 0.001).

## Discussion

The use of effective rhizobia was the first eco-friendly technology adopted to improve legumes resilience in hot climates. However, many *Rhizobium* species struggle to survive under harsh environmental conditions, leading to DNA and protein denaturation, which affects symbiotic functions (Favre and Eaglesham 1986; Räsänen and Lindström 1999; Gopalakrishnan et al. 2015). To address this, non-rhizobial bacterial endophytes isolated from legumes native to arid regions offer a promising strategy to improve the symbiotic performance of rhizobial strains, particularly those lacking ACC deaminase, when associated with a cool-season legume host under heat stress.

In our study, we found that rhizobial and non-rhizobial strains responded differently to both continuous heat stress and heat shock. Beghalem et al. (2017) previously reported that strains isolated from root nodules of legumes native to arid regions were resistant to 40 °C, suggesting a common physiological response to extreme environments. In contrast, our non-rhizobial endophytes, originating from root nodules of native legumes in arid habitats, exhibited a variety of responses to 40 °C. For example, while *P. turukhanskensis* (PS) was susceptible to 40 °C, *Ph. salinisoli* (PH) and *Starkeya* sp. (ST) had less pronounced effects. In addition, the *R. legumniosarum* (WT) strain, which was isolated from a cold-season legume, was severely affected by continuous heat stress but recovered from heat shock. The absence of ACC deaminase in rhizobial Δ*acdS* mutant (MT) strain had a significant impact on its response to continuous heat stress, indicating a role for ACC deaminase in heat stress response. Overall, these results suggest that bacterial physiological responses to high temperatures may constitute a genus-specific signature related to the functional conservation of stress-related genes.

The ACC deaminase enzyme plays a crucial part in plant-bacteria interactions by decreasing ethylene levels in plant tissues (Glick 2014). In the symbiotic rhizobium-legume relationship, ACC deaminase also enhances nodulation efficiency and competitiveness, especially under stress conditions (Tittabutr et al. 2015; Nascimento et al. 2016). As a result, the observed decrease in root growth and number of nodules in plants inoculated with the MT strain can be directly attributed to the mutant’s lack of ACC deaminase activity when compared to the WT strain. This finding is consistent with previous reports on rhizobium-pea symbiosis by (Ma et al. 2003) which demonstrated that the lack of ACC deaminase activity in *Rhizobium leguminosarum* strain impairs nodulation in pea plants under normal conditions. Furthermore, Savada et al. (2017) highlighted ethylene overproduction in peas as a common response to high temperatures. In this view, the decrease in MT symbiotic performance can be attributed to lower nodulation efficiency and higher ethylene levels in root tissues caused by heat stress.

Direct inoculation with rhizobia with high ACC deaminase activity (Nascimento et al. 2012a, b; Brígido et al. 2013) or co-inoculating legumes with specific plant growth-promoting bacteria possessing high ACC deaminase activity along with compatible rhizobial strains (Brígido et al. 2019a; Paço et al. 2020) are proven strategies for ensuring nodulation efficiency and promote legumes growth under various abiotic stresses. Here, combining the rhizobial MT strain with non-rhizobial endophytes that lack ACC deaminase activity under free-living conditions, improved MT-pea symbiosis and pea growth under heat stress. These findings suggest the presence of alternative molecular pathways that compensate for the MT strain’s lack of ACC deaminase activity. A potential explanation for these results might be the ability of these non-rhizobial endophyte strains to produce indole acetic acid (IAA). It is well known that IAA produced by bacteria promotes root growth by increasing the frequency of cell division and proliferation, hence improving root size, weight, number of branches, and nutrient absorption (Etesami et al. 2015; Santoyo et al. 2016). This could explain the increased root biomass in plants co-inoculated with non-rhizobial endophytes with the MT strain, potentially due to improved plant nutrition and water uptake, resulting in a reduced ethylene stress response released by pea roots and an increase in carbon and flavonoid exudation. Moreover, enhanced root development may also contribute to an increase in the number of rhizobial infection points, resulting in more nodules in plants co-inoculated with the MT strain and non-rhizobial endophytes consortia.

It should be noted that these non-rhizobial endophytes may colonize pea nodules, as has been observed in nodules of other legume species (Martínez-Hidalgo and Hirsch 2017). Although unable to induce nodulation, non-rhizobial endophytes possess genes for endophytic lifestyles and plant growth-promoting features (Tariq et al. 2014). When co-inoculated with rhizobia, they can enhance rhizobial nitrogen fixation and promote plant growth (Pastor-Bueis et al. 2021; Dhole et al. 2023). In this case, their IAA biosynthesis may foster the formation of larger nodules, allowing for a greater number of bacteroids compared to those produced only by the MT strain. Notably, combining the MT strain with *Phyllobacterium salinisoli* and *Starkeya* sp, commonly found in root nodules of various legume species (Zakhia et al. 2006; Rejii et al. 2014; León-Barrios et al. 2018), resulted in larger and heavier nodules than those formed by the mutant alone. The induction of exogenous IAA production inside nodules activates polyhydroxybutyrate (PHB) biosynthesis, which is crucial to bacteroid differentiation, thus enhancing the MT’s ability to colonize more cells and fix nitrogen efficiently (Imperlini et al. 2009; Duca et al. 2014).

In legumes, nitrogen fixation requires significant energy. Under stress conditions, like high temperatures, plants reduce photosynthesis to preserve energy for stress defense and interaction with rhizobia, ensuring a nitrogen source (Aranjuelo et al. 2007). Our study revealed decrease pea photosynthesis when inoculated with the MT strain compared to the wild-type, most likely due to heat stress combined by the MT’s lack of ACC deaminase activity. Plants inoculated solely with MT strain prioritize energy for symbiotic interaction and stress resistance over photosynthesis. However, co-inoculation of the MT strain with non-rhizobial endophytes improved chlorophyll biosynthesis, possibly through improved symbiosis and root development, leading to improved physiological status. Similar benefits have been observed in various plant species under different abiotic stresses when inoculated with similar endophytes (Egamberdieva et al. 2017; Ullah et al. 2017; Gupta and Pandey 2020; de Oliveira Lopes et al. 2022).

The degree of synergy among the MT strain, pea plants, and non-rhizobial bacterial endophytes seems to rely on the specific strains within the consortium. For instance, the ST + PS + PH consortium had a direct impact on the plant host, whereas the ST + PH consortium appeared to positively influence the MT-pea symbiosis. On the other hand, no benefits were observed in plants co-inoculated with the WT strain and any non-rhizobial bacterial endophytes consortia. Variation in plant-bacteria and bacteria-bacteria interactions can be attributed to plant genotype, bacterial species, and the interaction of heat stress and ACC deaminase activity.

Our results showed that heat stress significantly affected the phenolic content of pea root exudates, similar to studies in maize (Tiziani et al. 2022). Root exudates released into the rhizosphere contain some metabolites that are essential for plant-bacteria interactions. Several studies have shown that exudation patterns can be influenced by factors such as plant species, age, environmental stressors, and rhizosphere microbial communities (Olanrewaju et al. 2019; Vescio et al. 2021). Based on our findings, changes in the chemical composition of heat root exudates (HRE) did not affect IAA production but significantly influenced biofilm formation, which represents a key factor facilitating rhizobia attachment to plant roots and tissue colonization. The significant increase in biofilm formation observed in consortia including the MT strain and the non-rhizobial endophytes ST + PH might be attributed to variations in the phenolic composition of HRE, underscoring the importance of root metabolites in modulating bacteria-bacteria interactions. Vora et al. (2021) and Kumawat et al. (2019) reported similar effects, in which exposure to root exudates or dual combinations of bacterial strains increased biofilm formation and exopolysaccharide production. On the other hand, no significant improvement in biofilm production was observed when combining rhizobial WT strain with any non-rhizobial endophytic consortia, aligning with the absence of positive effects on pea growth and WT-pea symbiosis, which benefited from ACC deaminase activity. Several studies have highlighted the role of ACC deaminase-producing bacteria in providing plant resistance and adaptability to various abiotic stresses, including high temperatures (Mukhtar et al. 2020; Brunetti et al. 2021; Choi et al. 2022). Hence, the ACC deaminase enzyme from the WT strain might have helped maintain symbiotic interaction with pea plants by reducing ethylene stress levels in plant tissues, enhancing overall plant growth and heat stress tolerance compared to the MT strain. In the absence of ACC deaminase, mechanisms from non-rhizobial endophytes seem to be crucial in maintaining symbiosis and plant growth. However, with the WT strain, these mechanisms provide no additional benefit to WT-pea symbiosis. Overall, the molecular and biochemical mechanisms behind bacterial-bacteria interaction remain difficult to understand due to the interference of several physiological and environmental factors (Peix et al. 2015; Agudelo et al. 2023). Despite the high number of studies focusing on the characterization of the multiple aspects of quorum sensing in the rhizosphere, there’s still a lack in knowledge concerning the specific involvement of non-rhizobial endophytes in legume-*rhizobium* symbiosis (Menéndez and Paço 2020).

## Conclusions

Given the adverse effects of global warming on cool-season grain legumes and their symbiotic relationships, our study highlights the vulnerability of *Rhizobium*-pea symbiosis to heat stress when ACC deaminase functionality is absent. Conversely, we reveal the promising potential of non-rhizobial endophytes sourced from legumes native to arid regions in enhancing the symbiotic performance of rhizobia lacking ACC deaminase activity under heat stress conditions. Additionally, our data illustrate the impact of heat stress on the phenolic composition of root exudates and its implications for bacteria-bacteria interaction. Our findings also suggest that compatible rhizobia expressing ACC deaminase alone serve as effective bioinoculants for cool-season pea plants cultivated in regions prone to heat waves, such as the Mediterranean. However, in the absence of ACC deaminase in compatible pea microsymbionts, combining other endophytes is necessary to achieve better symbiotic and pea growth outcomes.

### Electronic supplementary material

Below is the link to the electronic supplementary material.

Supplementary Material 1

## Data Availability

No datasets were generated or analysed during the current study.
