# Supplementary material for: Enhancing Pisum sativum growth and symbiosis under heat stress: the synergistic impact of co-inoculated bacterial consortia and ACC deaminase-lacking Rhizobium
Source: Arch Microbiol. 2024 Apr 4;206(5):203. doi: 10.1007/s00203-024-03943-3 (PMC10995081; doi:10.1007/s00203-024-03943-3)
Supplement: Supplementary file 1 — Supplementary Material 1 [file 203_2024_3943_MOESM1_ESM.pdf]

Enhancing *Pisum sativum* growth and symbiosis under heat stress: the synergistic impact of co-inoculated bacterial consortia and ACC deaminase-lacking *Rhizobium*

Roukaya Ben Gaied [1,2], Imed Sbissi [1], Mohamed Tarhouni[1], and Clarisse Brígido [3,*]

[1]Laboratory of Pastoral Ecosystems and Promotion of Spontaneous Plants and Associated Micro-Organisms, Institute of Arid Lands, University of Gabes, Medenine 4119, Tunisia.

[2]MED – Mediterranean Institute for Agriculture, Environment and Development, Universidade de Évora, Pólo da Mitra, Ap. 94, 7006-554 Évora, Portugal.

[3]MED – Mediterranean Institute for Agriculture, Environment and Development & CHANGE – Global Change and Sustainability Institute, Institute for Advanced Studies and Research, Universidade de Évora, Pólo da Mitra, Ap. 94, 7006-554 Évora, Portugal.

***Correspondence: ccb@uevora.pt**

Table S1. Phenolic acids and flavonoids composition of *P. sativum* root exudates under control and heat stress conditions determined by Liquid chromatography-mass spectrometry (LC-MS).

**Compound**	**Control** (ppm/mg)	**Heat** (ppm/mg)
Quinic acid	7.577 ±0.330 [a]	1.633 ±0.046 [b]
Gallic acid	0.055 ±0.003 [a]	0.028 ±0.001 [b]
Protocachuic acid	1.350 ±0.016 [a]	0.094 ±0.007 [b]
Cholorogenic acid	0.126 ±0.002 [a]	0.080 ±0.003 [b]
Caffeic acid	22.761 ±0.114 [a]	0.349 ±0.008 [b]
1,3-di-O-caffeoyquinic acid	47.696 ±4.460 [a]	33.311 ±0.611 [b]
Syringic acid	8.322 ±0.232 [a]	0.534 ±0.005 [b]
Epicatechin	2.586 ±0.070 [a]	0.700 ±0.004 [b]
Rutin	0.802 ±0.050 [a]	-
Naringin	2.735 ±0.198 [a]	-
Quercetrin (quercetin-3-o-rhamonoside)	1.344 ±0.017 [a]	-
Rosmarinic acid	6.230 ±0.075 [a]	-
Salviolinic acid	0.626 ±0.015 [a]	-
Apegenin-7-o-glucoside	-	0.179 ±0.0002 [a]
Trans cinnamic	-	0.111 ±0.003 [a]

(-) Absence of the compound; Data represent means and standard deviation of three independent replicates under each condition; Different letters indicate statistical significance ($p < 0.01$).
